# Reducing Patient Loneliness With Artificial Agents: Design Insights From Evolutionary Neuropsychiatry

**DOI:** 10.2196/13664

**Published:** 2019-07-08

**Authors:** Kate Loveys, Gregory Fricchione, Kavitha Kolappa, Mark Sagar, Elizabeth Broadbent

**Affiliations:** 1 Department of Psychological Medicine The University of Auckland Auckland New Zealand; 2 Benson-Henry Institute for Mind Body Medicine Massachusetts General Hospital Boston, MA United States; 3 Department of Psychiatry Harvard Medical School Boston, MA United States; 4 Auckland Bioengineering Institute The University of Auckland Auckland New Zealand

**Keywords:** loneliness, neuropsychiatry, biological evolution, psychological bonding, interpersonal relations, artificial intelligence, social support, eHealth

## Abstract

Loneliness is a growing public health issue that substantially increases the risk of morbidity and mortality. Artificial agents, such as robots, embodied conversational agents, and chatbots, present an innovation in care delivery and have been shown to reduce patient loneliness by providing social support. However, similar to doctor and patient relationships, the quality of a patient’s relationship with an artificial agent can impact support effectiveness as well as care engagement. Incorporating mammalian attachment-building behavior in neural network processing as part of an agent’s capabilities may improve relationship quality and engagement between patients and artificial agents. We encourage developers of artificial agents intended to relieve patient loneliness to incorporate design insights from evolutionary neuropsychiatry.

## Introduction

Artificial agents, such as robots and chatbots, are currently being developed to provide companionship and assist patients with health care needs [1,2]. The purpose of this paper is to argue that incorporating mammalian attachment-building behavior into agent design may increase agent effectiveness. First, the paper presents loneliness as a growing public health issue and discusses the promise of social support interventions for treating loneliness. The paper then describes recent social support interventions delivered by artificial agents. Finally, the paper presents insights from evolutionary neuropsychiatry and describes mammalian attachment-building behaviors that may be included in artificial agents to promote patient engagement.

## Loneliness, Social Connection, and Health

Loneliness is a widespread global health issue that approximately affects a third of people in industrialized countries [3]. Loneliness refers to a subjective state of social isolation in which the individual perceives a mismatch between ideal and actual social relations [4]. According to a recent report by the Jo Cox Commission, almost a quarter of parents with young children felt *always or often lonely*, more than a third of those aged over 75 years reported feelings of loneliness out of their control, and, in one year, more than 4000 children reported feeling *unbearably lonely* in the United Kingdom alone [5]. Hospitalized patients are at a particularly high risk of loneliness [6].

Although the occasional feeling of loneliness acts as an adaptive signal to seek social interaction, chronic loneliness can be detrimental to health. Loneliness increases mortality risk by 32% [7], a degree comparable with smoking 15 cigarettes daily [8]. Loneliness elevates the risk of many morbidities, including stress-related conditions (coronary heart disease, stroke, and high blood pressure) [9], and psychiatric illnesses (major depression, psychosis, and suicide) [10]. Loneliness places a significant burden on health care systems through increased health care utilization [11], costing an estimated additional US $6.7 billion per year for older adults alone [12].

Social connection refers to quality social relations characterized by perceived belongingness and closeness [13]. Greater social connection is needed to counteract the significant detrimental effects of loneliness on health and longevity and relieve the burden on health care systems.

## Social Support Interventions

Many researchers are testing social support interventions as a means to improve social connection and reduce loneliness. Social support refers to a functional exchange of emotional, informational, or practical aid between individuals [14]. One strategy to provide social support is via community-level interventions. An example is the Campaign to End Loneliness, which promotes small actions of social connection between strangers in the United Kingdom. Other examples include the Reconnections Service (which links older adults to social activities), computer skills training for older adults to engage with others on the Web [15], peer support [14], and altering the environment to be more conducive to social interaction by providing pedestrian-focused public spaces [16].

One way that social support interventions protect health is by buffering against the impact of stress on the body [17]. Chronic stress increases inflammation [18], lowers heart rate variability [19], and impairs immune response [20], which increase the risk of physical and mental morbidities [18,20,21]. Social support can reduce sympathetic nervous system activation [22], increase oxytocin secretion, and suppress cortisol release [23], which reduce the impact of chronic stress [17]. Social support can also indirectly benefit health through the provision of health information, treatment adherence encouragement, or practical support [24].

Interventions to improve social support have generally been shown to have benefits for health and well-being, including reduced stress [23], lower anxiety and depression [25], decreased alcohol consumption [26], and improvements in wound healing [27], treatment adherence [28], myocardial infarction recurrences, and mortality [29]. A systematic review of 100 studies concluded that social support interventions generally provide health benefits irrespective of the type of support provided in the intervention and whether interventions were delivered to an individual or group or were professionally led or provided by peers [14]. However, the effectiveness of social support may be impacted by components of relationship quality between the patient and support provider, such as social closeness. Social closeness refers to a relationship quality where partners regularly engage in intimate behaviors such as support, self-disclosure, and shared activities [30]. Characteristics of the partner can also affect how close a connection is formed; these include perceived familiarity [31], warmth [30], and empathic accuracy [32].

## Social Support From Artificial Agents

Traditional social support interventions may not always be available or desirable. In some situations, faced with the absence of human connection, artificial agents may provide support akin to human social support to benefit health. Artificial agents in health care may act as transitional objects that help patients to cope with feelings of loneliness and the depressive anxiety that often accompanies severe illness and end of life experiences [33,34]. Artificial agents have been shown to reduce feelings of loneliness [35], as well increase interrelatedness, either through direct interactions with the agent or by triggering conversations between humans that might not have otherwise occurred [1].

In addition to social benefits, artificial agents have been shown to exert positive effects on physical and mental health. Paro (Intelligent System Co, Ltd, Japan), a companion robot in the form of a fluffy baby harp seal, has been shown to improve mood [36] and reduce depression symptoms for people with dementia [37]. Paro was designed with big eyes and soft fur to encourage users to feel affectionate toward it like a real baby animal. iRobi (Yujin Robot Co, Ltd, Korea), a robotic homecare companion, significantly improved medication adherence and rehabilitation exercise frequency for patients with chronic obstructive pulmonary disease through providing information and reminders [38]. Conversational agents have demonstrated benefits for mental health, such as reduced depression and anxiety symptoms [2], and other forms of artificial companionship are being explored in the context of health, including Alexa (a virtual voice assistant made by Amazon) [39].

The characteristics of an artificial agent providing social support may affect the success of an intervention. If an agent closely models realistic human interactions, this may increase patients’ willingness to develop social closeness with the agent [40]. A very high degree of human likeness improves perceptions of agents’ social characteristics [41], and appropriate use of human-like verbal and nonverbal relational cues improve an agent’s relationship with users [42]. The most important behaviors for developing social closeness and support between humans and artificial agents remain to be determined. Behaviors from the natural world may provide some promising design strategies.

## Design Insights From Evolutionary Neuropsychiatry

We propose that evolutionary neuropsychiatry offers important insights for the design of artificial agents to provide complementary support meant to be additive and not substitutive to human support. Aspects of mammalian brain evolution that enabled social attachments provide direction to engineers as to the necessary internal structure, processes, and output required of systems to appropriately elicit attachment in a way that maximally supports human users.

MacLean studied aspects of brain evolution across reptiles and mammals and found that for mammalian brain evolution, specifically, particular structures evolved that enabled the mammalian behavioral triad [43]. The mammalian behavioral triad comprises maternal nurturance, the separation call, and social play. These behaviors serve the purpose of strengthening social attachment, which is the mammalian survival strategy.

For the mammalian behavioral triad to be possible, as well as the ability for mammals to select attachment or separation as a response to environmental objects, certain brain structures and loops evolved. The *protolimbic* loop evolved to manage attachment to food and reproductive objects [33]. Two primordial moieties assist the protolimbic loop: the hippocampocentric moiety specifies where the organism is located in relation to objects in the environment, whereas the olfactocentric moiety classifies objects that are located within the environment [33]. The mammalian brain evolved in such a way that these 2 moieties converged in paralimbic cortical zones, namely, the anterior cingulate cortex (ACC), the medial orbitofrontal cortex (mOFC), and the anterior insula. This created a terminal zone for a *paralimbic* basal ganglia thalamocortical circuit. In the paralimbic loop, the ACC works with the mOFC to synthesize and make emotional and cognitive classifications of input to inform decisions about whether to separate from or attach to an object. The convergence of these areas created a response selecting area which can be traced to the primary separation challenge attachment solution paradigm [33]. This paradigm indicates requirements for mammals to attach not only to sources of metabolic energy and reproductive success but also to sources of social support for survival. The evolution of these particular structures and their convergence in the mammalian brain provided the basis for social attachment, both within and between mammalian species.

**Figure 1:**
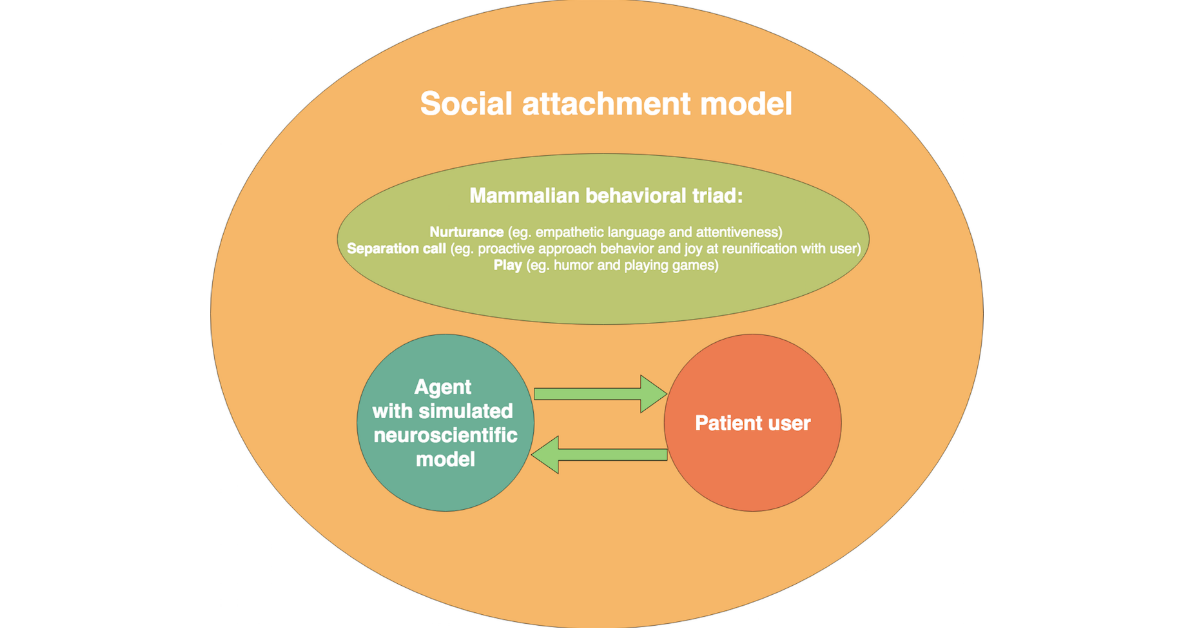
Model of behavior that may increase patient engagement with artificial agents according to evolutionary neuropsychiatry.

We propose that artificial agents designed for social support provision be created with internal models of the neural structures, processes, and output that evolved to enable genuine social attachment between mammalian species. This involves creating agents with environmental sensors, classifiers for incoming data on emotion and attachment behavior, and interaction memory with a user, along with the behavioral capacity to produce the mammalian behavioral triad. For example, nurturance could be shown by attentiveness and use of empathetic language; the separation call could be shown through the proactive arrangement of another meeting in the future; and play could be demonstrated through the use of humor. A model of social attachment between artificial agents and patients is shown (Figure 1). The simulation of biological processes necessary for producing such behaviors in artificial agents is complex, but substantial progress has been made toward linking neuroscience models with computer graphic interfaces for creating life-like facial expressions during interactions [40].

Ideally, patients and agents should form a reciprocal attachment over time with repeated interactions. It is an aspiration that future artificial agents in health care may have embedded the capacity to produce an efficacious facsimile of social attachment, which may enhance the potency of an agent’s social support, reduce patient loneliness, and improve patient engagement with care.

Although we advocate that design of artificial agents be inspired by the evolutionary neuropsychiatry of social attachment, we express the caveat that these agents would serve only as adjuvant social support boosters and would not be designed as substitutive for genuine human attachments. We also acknowledge that the design of artificial agents intended for a high degree of human interaction is a complex, sensitive issue that requires multidisciplinary discussion by diverse stakeholders and demographic groups, particularly in relation to ethics and evaluation [44,45]. Further consideration of safeguards embedded into agent design and implementation, as well as ongoing evaluations using validated metrics, is necessary to ensure social connection with artificial agents is beneficial for patients.

## Abbreviations

ACC: anterior cingulate cortex
mOFC: medial orbitofrontal cortex
